# Increasing the vigour and sustainability of Lei bamboo (*Phyllostachys violascens*) plantations under mulching conditions by aeration through buried subsurface pipes

**DOI:** 10.3389/fpls.2026.1877299

**Published:** 2026-08-28

**Authors:** Shixin Deng, Jie Meng, John L. Innes, Yichang Guo, Renyi Gui

**Affiliations:** 1 State Key Laboratory for Development and Utilization of Forest Food Resources, Zhejiang A&F University, Hangzhou, China; 2 Bamboo Industry Institute, Zhejiang A&F University, Hangzhou, China; 3 Department of Forest Resources Management, Faculty of Forestry, University of BritishColumbia, Vancouver, BC, Canada

**Keywords:** aeration, leaf photosynthetic rate, mulching, root vitality, soil enzymes

## Abstract

The sustainable production of bamboo shoots is jeopardized by current management practices, including the use of winter mulches and the application of large quantities of nitrogen fertilizer. Many bamboo plantations exhibit premature aging and reduced shoot yields, primarily linked to oxygen deprivation in the rooting zone from mulching practices but also associated with soil acidification. The mulching is undertaken to raise soil temperatures, encouraging earlier shoot growth, but it leads to a decline in shoot production and the practice is clearly unsustainable. We examined whether the use of aeration in Lei bamboo plantations could improve the soil environment, and whether any observed soil improvements were associated with improved plant vigour. We used four field treatments (mulching without aeration, M; mulching with aeration, MA; no mulching with aeration, A; and no mulching without aeration, CK). The effects of aeration were assessed in terms of soil enzyme activity, root growth, and leaf photosynthesis. Aeration (MA and A) consistently improved key metrics compared to non-aerated treatments (M and CK). Soil enzyme activities were markedly affected: In June, catalase activities in the soils of the aerated groups (A and MA) were 22.35% and 37.25% higher, respectively, than those in non-aerated groups. Urease activities were 28.64% (A vs. CK) and 124.08% (MA vs. M) higher, respectively, and phosphatase activities were 208.80% and 131.71% higher, respectively. By October, root vitality in aerated groups was 61.90% (A vs. CK) and 142.67% (MA vs. M) higher, respectively, compared to non-aerated groups. The leaf photosynthetic rate in the A treatment was 114.48% higher than in the CK treatment and 88.31% higher in the MA treatment than in the M treatment. The results demonstrate that soil aeration leads to the more sustainable production of bamboo shoots.

## 1 Introduction

Bamboo has long been cultivated as it has many uses, including fiber, bio-based materials, construction, paper, food, and traditional medicine (Peng et al., 2013; Liese and Kohl, 2015). As such, bamboo has been helping to meet global challenges including the alleviating rural poverty, unsustainable resource use and climate warming. Significant areas of land in China, southeast Asia and South Asia are devoted to its cultivation, and the area under cultivation of South and Central America is growing. However, bamboo requires active management and, in the absence of this, its vigour and productivity will decline. Because of its long history of cultivation, management systems have evolved aimed at enhancing the production of particular features, such as edible shoots or large-diameter culms. However, it is becoming increasingly apparent that some of these management practices are unsustainable, detracting from what would otherwise be considered as a green product.

The intensive production of bamboo shoots from species such as *Phyllostachys edulis*, *Phyllostachys violascens* and *Dendrocalamus latiflorus* commonly involves organic mulching techniques, along with the addition of heat-producing substances and insulation materials aimed at increasing late winter and early spring soil temperature (Fang et al., 1994). This results in the advancement of bamboo shoot emergence to before the Chinese Spring Festival, thereby enhancing economic benefits because of the high price premiums that these early shoots fetch (Fan et al., 2021, 2025). However, long-term mulching management can adversely affect plant growth by impeding oxygen diffusion between the soil and atmosphere, leading to hypoxic stress (Xu et al., 2017; Qian et al., 2021). This can lead to the phenomenon known as floating, with the rhizomes floating (Xu et al., 2017). After 3–4 years of continuous mulching management, underground rhizomes begin to degrade and bamboo shoot quality declines (Gui et al., 2013; Chen et al., 2014). While many mechanisms have been proposed for the decline in production, including late removal of the mulch (Zhou et al., 1999), phosphorus accumulation in the soil (Jiang et al., 2000), nutritional imbalances (Yu et al., 2001), phenolic acid leaching from the mulch (Zheng R, 2006) and increased aluminum concentrations due to acidification (Gui et al., 2013), it is now accepted that insufficient soil oxygen under the standard continuous mulching management model may be one of the main causes of the observed degradation of bamboo forests (Xu et al., 2017).

Soil aeration is a critical factor influencing plant growth. Insufficient aeration reduces soil enzyme activity, decreases the availability of certain nutrients (including phosphorus, nitrogen, and iron), lowers soil fertility, and ultimately affects plant growth and development. Soil urease, a key enzyme in the soil nitrogen cycle, is produced by urea-decomposing microorganisms and hydrolyzes urea to produce ammonia, thereby supplying nitrogen to plants (Mekonnen et al., 2021). Catalase, another soil enzyme, catalyzes the decomposition of hydrogen peroxide into oxygen and water, mitigating its toxicity to plants, and is an important enzyme for evaluating soil fertility (Trasar-Cepeda et al., 1999). Soil phosphatase, an extracellular enzyme secreted by plants and microorganisms, hydrolyzes organic phosphorus in the soil to provide available phosphorus for plants, with its activity directly impacting the organic phosphorus cycle in soils (Gao et al., 2022). These soil enzymes respond to soil aeration. The activity of catalase and urease in the rhizosphere soil of greenhouse-grown melons increased significantly when air was injected into the soil (Li et al., 2015). This form of aeration has also been shown to enhance microbial abundance and enzyme activity in soil with tomatoes (Sun et al., 2024) and peaches (Xiao et al., 2015), and ultimately increasing crop yields. Rhizosphere aeration also enhances root activity, improving plant water and nutrient uptake capacity (Niu et al., 2011). Aeration increased the root activity in potted maize, promoting plant growth and development (Guo and Niu, 2010). Similarly, rhizosphere aeration enhanced root metabolism, influencing root cap growth in cucumbers (*Cucumis sativus*) (Li et al., 2008, 2009).

While increased aeration can boost productivity on many crops, insufficient soil oxygen inhibits aerobic respiration, reducing the energy supply required for plant growth and development, thereby impacting root activity and leaf photosynthesis (Horchani et al., 2008). There are numerous studies demonstrating how soil compaction can reduce the availability of oxygen in the soil, as well as impeding drainage. For example, Johansen et al. (2014) reported that poor soil drainage and poor aeration, caused by mechanical compaction, impaired the root growth of potatoes and carrots, while improving soil aeration significantly increased crop yields. Under hypoxic conditions, plant roots undergo anaerobic respiration, producing malondialdehyde (MDA), which promotes the synthesis of reactive oxygen species (ROS). ROS are both universal markers of oxidative stress and signaling molecules indicative of stress adaptation responses (Jia et al., 2019). However, excessive ROS production can damage cellular structures, nucleic acids, and proteins (Liu et al., 2018). For example, a decrease in oxygen concentration in the rhizosphere of *Beta vulgaris* led to a reduced capacity for ROS scavenging, resulting in root oxidative damage (Dong et al., 2022). Thus, maintaining a balance between ROS production and scavenging is critical for plant survival under hypoxic stress (Zeng et al., 2020).

Metabolic and growth changes caused by root hypoxia can be transmitted to the leaves, leading to a decline in plant photosynthesis and ultimately affecting plant yield. Photosynthesis is a critical process in the growth and development of all green plants, providing the chemical energy necessary for metabolism (Ashraf and Harris, 2013). Under low-oxygen stress, the activity of photosynthetic enzymes decreases, resulting in reduced photosynthetic capacity and diminished plant vigor (Ashraf and Harris, 2013). For example, under conditions of root hypoxia, the photosynthesis of tomatoes was limited (Mauro et al., 2020), and growth and development were adversely affected.

Long-term mulching can lead to poor soil aeration in bamboo plantations, adversely affecting the soil microbial community (Wu et al., 2023; Zhang L.P. et al., 2024). Depending on the pattern of application, mulching can also lead to increased soil CO_2_ emissions (Li et al., 2025), although regular winter mulching can result in increasing levels of soil organic carbon over time (Zhuang et al., 2011). The reduced oxygen levels in the soil result in the floating and aging of bamboo rhizomes, a reduction in leaf area index, and even flower mortality in Lei bamboo (*Phyllostachys violascens*) forests (Zhang et al., 2001). Improving soil aeration under poorly aerated conditions could play a critical role in promoting root and plant growth, as well as increasing yield. (Qian et al. 2022a, b) and Mbukwa et al. (2023a, b) have demonstrated that soil aeration treatment in *Phyllostachys praecox* (now recognized as *Phyllostachys violascens*) effectively alleviates soil acidification, increases oxygen levels and soil nutrient availability, and significantly enhances the diversity and richness of soil bacteria and fungi, thereby promoting culm yield. Simultaneously, aeration treatment increases the concentration of indole-3-acetic acid (IAA), while reducing the levels of gibberellin, ethylene, abscisic acid, and the activity of various anaerobic enzymes (Gao et al., 2023). Such results emphasize the importance of environmental drivers in determining the quality of bamboo shoots (Qian et al., 2025). Although progress has been made in research on bamboo forest aeration, systematic studies on the effects of oxygen enrichment on site conditions and growth remain relatively limited.

Lei bamboo plantations also experience excessive nitrogen fertilizer application, a factor leading to their acidification and subsequent deterioration in stand health and productivity (Liu et al., 2010; Gui et al., 2013; Wu et al., 2023). The application of nitrogen to bamboo plantations is known to have many effects, including reducing the soil organic content (Chu et al., 2024a, b). However, disentangling the interactive effects of nitrogen fertilizer applications and mulch applications remains challenging.

This study focused on Lei bamboo, an economically significant bamboo species extensively cultivated in Zhejiang, Anhui, and Jiangsu provinces of China (Cai et al., 2019; Hou et al., 2022). It examined the effects of soil aeration on the bamboo, with buried pipe aeration technology being used to increase soil oxygen concentration. We hypothesized that any responses would be evident in changes in soil enzyme activity, root activity, and leaf photosynthesis. This information could be used to develop methods to reverse the observed decline in bamboo plantations and provide more sustainable methods of bamboo shoot production.

## 2 Materials and methods

### 2.1 Overview of the experimental site

The experimental site was located at the Lei Bamboo Forest Base of Zhejiang A&F University in Panmugang, Taihu Source Town, Lin’an District, Hangzhou City, Zhejiang Province, China (119°37′40″E, 30°11′47″N). The area features a north subtropical monsoon climate, and is predominantly characterized by hilly and mountainous terrain. The average annual precipitation is 1,614 mm, spread over 158 rainy days, and the frost-free period lasts 237 days. The brown soil at the site developed from siltstone parent material and exhibited a medium to heavy loam texture with weak acidity (pH 6.3). It contained 2.5 g/kg organic matter and 1.7 g/kg total nitrogen. The available nitrogen, available phosphorus, and readily available potassium levels were 149.0 mg/kg, 317.4 mg/kg, and 186.3 mg/kg, respectively. The standing bamboo density was 15,000 culms per hectare, with an average diameter at breast height (DBH) of 3.89 cm. The age structure of the culms in the Lei bamboo forest, represented as 1-, 2-, and 3-year-old culms is 1:1.89:0.58. Throughout the entire experimental period, uniform cultivation practices were applied to all treatments. Compound fertilizer was applied at a rate of 0.60 t/ha in May, September, and November, with a nutrient ratio of N:P_2_O_5_:K_2_O = 16%:16%:16%.

### 2.2 Experimental design

Four treatment types were established in the bamboo forest: mulching without aeration (M); mulching with aeration (MA); no mulching with aeration (A); and no mulching without aeration (CK). A randomized complete block design was employed, with each treatment assigned to three experimental plots (10 m × 3 m) randomly selected within the study area under relatively uniform site conditions, resulting in a total of 12 experimental plots across the four treatments. The experiment was established in December 2022, with dynamic monitoring of all parameters commencing in January 2023.

**Figure 1:**
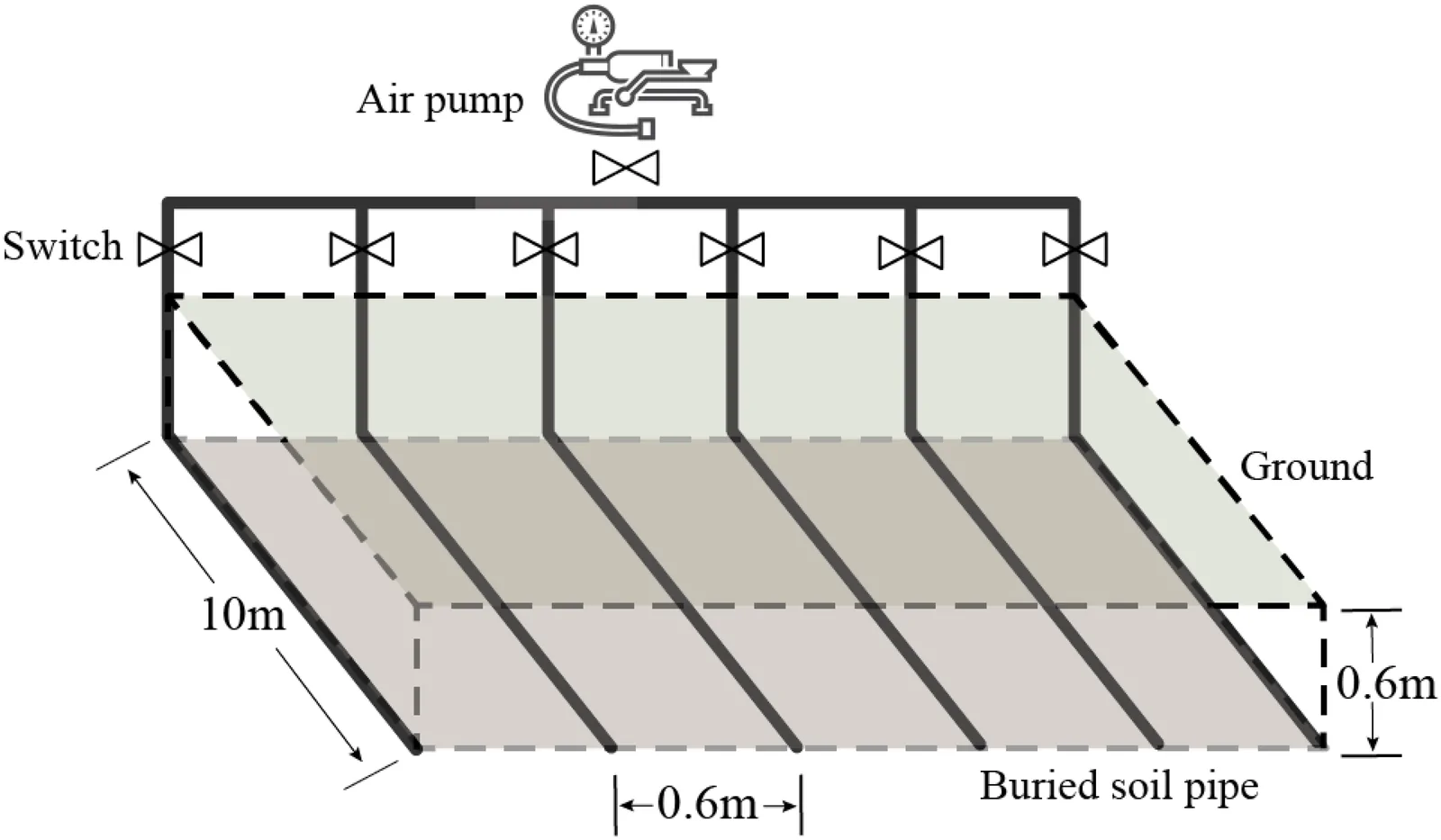
Schematic showing the layout of the buried soil pipes under mulched Lei bamboo plantations.

As the underground rhizome system of Lei bamboo is primarily distributed at a depth of 20–30 cm (Chen, 2013; Tong et al., 2020), we used a drilling machine to construct horizontal tunnels at a depth of 60 cm in each planting plot to avoid damaging the rhizome roots and ensure adequate oxygen supply. The tunnels were spaced 60 cm apart. Plastic PVC aeration pipes with an outer diameter of 21 mm and a wall thickness of 1 mm were laid through these holes and connected sequentially. Small holes with a diameter of 0.2 mm were drilled into the pipe walls at 30 cm intervals to allow air to escape. The pipes were used solely for aeration. The main aeration pipes in each plot were connected to an air pump, which pushed ambient air into the soil through the pipes, using a compressor (Quanzhou Jinba, V-1.05/12.5, pressure 12 kg) (**Figure 1**). Aeration was conducted at 7:30 and 16:30 daily, with each session lasting 40 minutes and aeration ran for 20 min, paused for 20 min, repeated twice per session.

The mulching in the Lei bamboo forest plots was carried out in accordance with local farmers’ traditional practices. In December, the ground surface of the bamboo forest was first covered with 10–15 cm of wheat residues left after threshing. This was followed by an additional 10–15 cm layer of rice husks as insulation. While this level of mulching is standard practice in intensive bamboo management, the 20–30 cm deep mulch is much more than the standard mulch of ca. 10 cm that is widely used in soil treatments (Masciandaro et al., 2004).

### 2.3 Measurement indicators and methods

#### 2.3.1 Soil oxygen level

Soil oxygen concentrations were measured using a fiber-optic oxygen meter (Firesting O_2_, Pyro Science, Germany). The instrument was calibrated prior to use with a two-point calibration, using air-saturated water (21% O_2_) and a saturated sodium sulfite solution (0% O_2_). The probes were vertically inserted into the soil to burial depths of 10, 20, 30, and 40 cm directly above the aeration pipes. After backfilling and compacting the soil, measurements were taken following a one-week stabilization period. For each treatment, three replicate plots were established, with five replicate measurements per plot, averaged for analysis.

#### 2.3.2 Soil temperature

Soil temperature was measured using a soil temperature instrument (LTW, China). A temperature probe was installed horizontally, perpendicular to the aeration pipe at a depth of 30 cm and a distance of 30 cm. This depth corresponds to the 20–30 cm soil layer where Lei bamboo rhizomes are concentrated. After backfilling and compacting the soil, measurements were taken following a one-week stabilization period. For each treatment, three replicate plots were established, with five repeated measurements conducted in each plot.

#### 2.3.3 Soil enzyme activity

Using the five-point sampling method, rhizomes from the 20–30 cm soil layer were excavated, and large soil clumps were shaken off. The soil adhering to the rhizomes was brushed off, thoroughly mixed, and treated as a single composite sample. Each treatment had 3 biological replicates, with five soil samples taken. Catalase activity was measured using the potassium permanganate titration method (Li, 2000), urease activity was determined using the indophenol blue colorimetric method (Yang, 1988), and phosphatase activity was analyzed using the disodium phenyl phosphate colorimetric method (Guan, 1987).

#### 2.3.4 Root activity

Using a five-point sampling method, 15 segments of one-year-old fine roots attached to rhizomes from the 20–30 cm soil layer were carefully collected from each treatment plot. The rhizosphere soil was gently shaken off, and healthy roots were selected. These roots were thoroughly cleaned with deionized water, blotted dry with absorbent paper, and their activity was measured using the TTC (2,3,5-Triphenyltetrazolium chloride) method (Zheng B, 2006). Each treatment had 3 biological replicates.

#### 2.3.5 Superoxide dismutase and peroxidase activity

Six bamboo plants were randomly selected from each of the three experimental plots for each treatment, totaling 18 plants. Roots were collected, flash-frozen in liquid nitrogen, and stored at -80 °C for later analysis. Superoxide dismutase (SOD) activity was determined using a SOD test kit developed by the Nanjing Jiancheng Bioengineering Institute. Peroxidase (POD) activity was measured using the guaiacol method (Chen and Li, 2016).

#### 2.3.6 Malondialdehyde content

Six bamboo plants were randomly selected from each of the three experimental plots for each treatment, totaling 18 plants. Roots were collected, flash-frozen in liquid nitrogen, and stored at -80 °C for analysis. MDA content, a marker of oxidative stress, was determined using the thiobarbituric acid method (Lv, 2016).

#### 2.3.7 Leaf photosynthesis

Using a Li-6400 portable photosynthesis measurement system, net photosynthetic rate (P*n*), transpiration rate (T*r*), and intercellular CO_2_ concentration (Ci) were measured between 9:00 and 11:00 a.m. on sunny, windless days. Six bamboo plants were randomly selected from each of the three experimental plots for each treatment, totaling 18 plants. For each plant, a mature leaf from the top of the first branch was chosen. Saturated light intensity was set at 1000 μmol/m²/s, and CO_2_ concentration was maintained at 400 μmol/mol.

#### 2.3.8 Soluble sugar and soluble protein content in leaves

Soluble sugar content was determined using the ultraviolet spectrophotometry method (Chen and Li, 2016); Soluble protein content was measured using the Coomassie Brilliant Blue staining method (Urrestarazu and Mazuela, 2005).

### 2.4 Data processing and analysis

Data were analyzed statistically using SPSS 20.0 software (IBM, NY, USA). All values were expressed as the mean ± standard deviation (SD). The significance of differences among treatment data was tested using Duncan’s multiple range test (*p* < 0.05) for mean separation. Graphs were prepared using Origin software.

## 3 Results

### 3.1 Effects of soil aeration on soil oxygen levels and soil temperature

**Figure 2:**
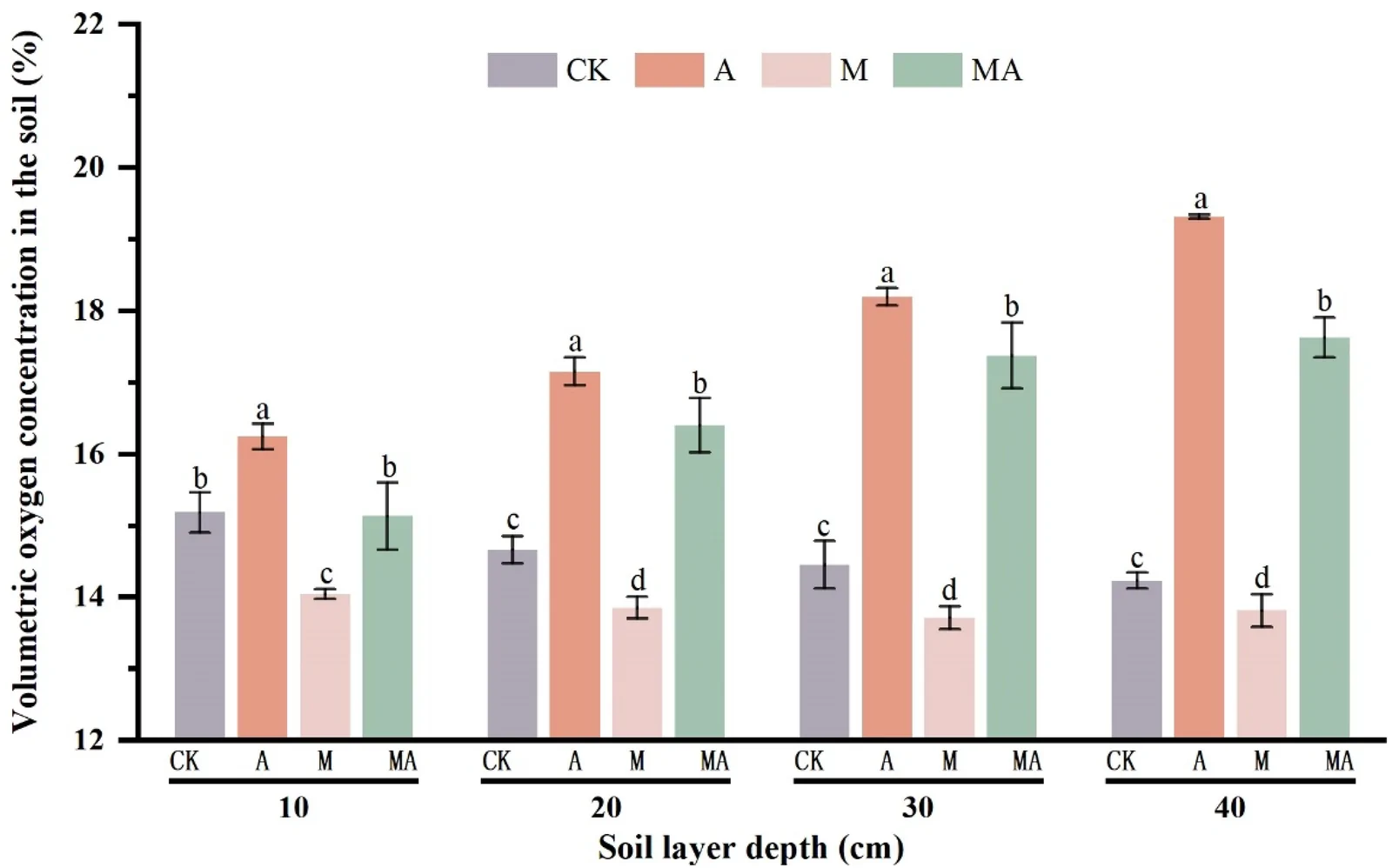
Soil oxygen content in vertical layer under different treatments. The error bars represent the standard deviation (SD), n = 15. Different letters indicate significant differences between different treatments at the same depth (*p* < 0.05).

The differences in oxygen concentration in the different soil layers were significant under the different treatments (**Figure 2**). The oxygen concentration in the soil under aerated treatment was higher than that under non-aerated treatment, indicating that the pipe-buried aeration method functioned as intended. As might be expected, the oxygen concentrations were highest at 20–30 cm (rhizome zone) in aerated soils, decreasing toward the surface and 40 cm depth, whereas in the soils without aeration, the highest oxygen levels were found at 10 cm depth, and were lower at 20, 30 and 40 cm depth. This reflects the penetration of air downwards from the surface of the soil.

**Figure 3:**
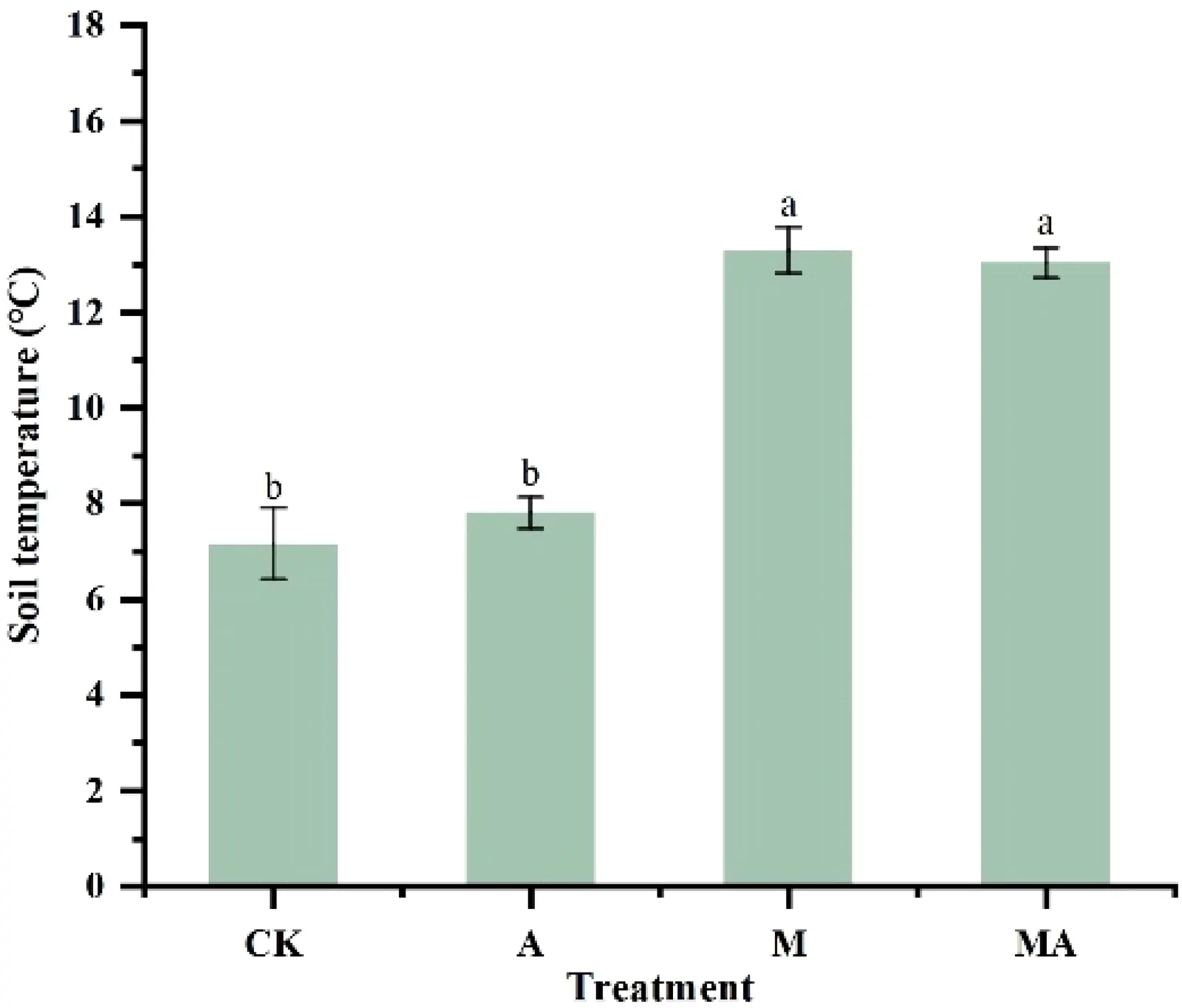
The effect of soil aeration on soil temperature. The error bars represent the standard deviation (SD), n = 15. Different letters indicate significant differences between different treatments (*p* < 0.05).

Temperature is one of the primary environmental factors influencing bud-to-shoot transformation. There were no significant differences in soil temperature among the treatments across all sampling dates, regardless of mulching or aeration, indicating that the buried pipe ventilation method did not exert a noticeable impact on soil temperature (**Figure 3**).

### 3.2 Effects of soil aeration on soil enzyme activity

**Figure 4:**
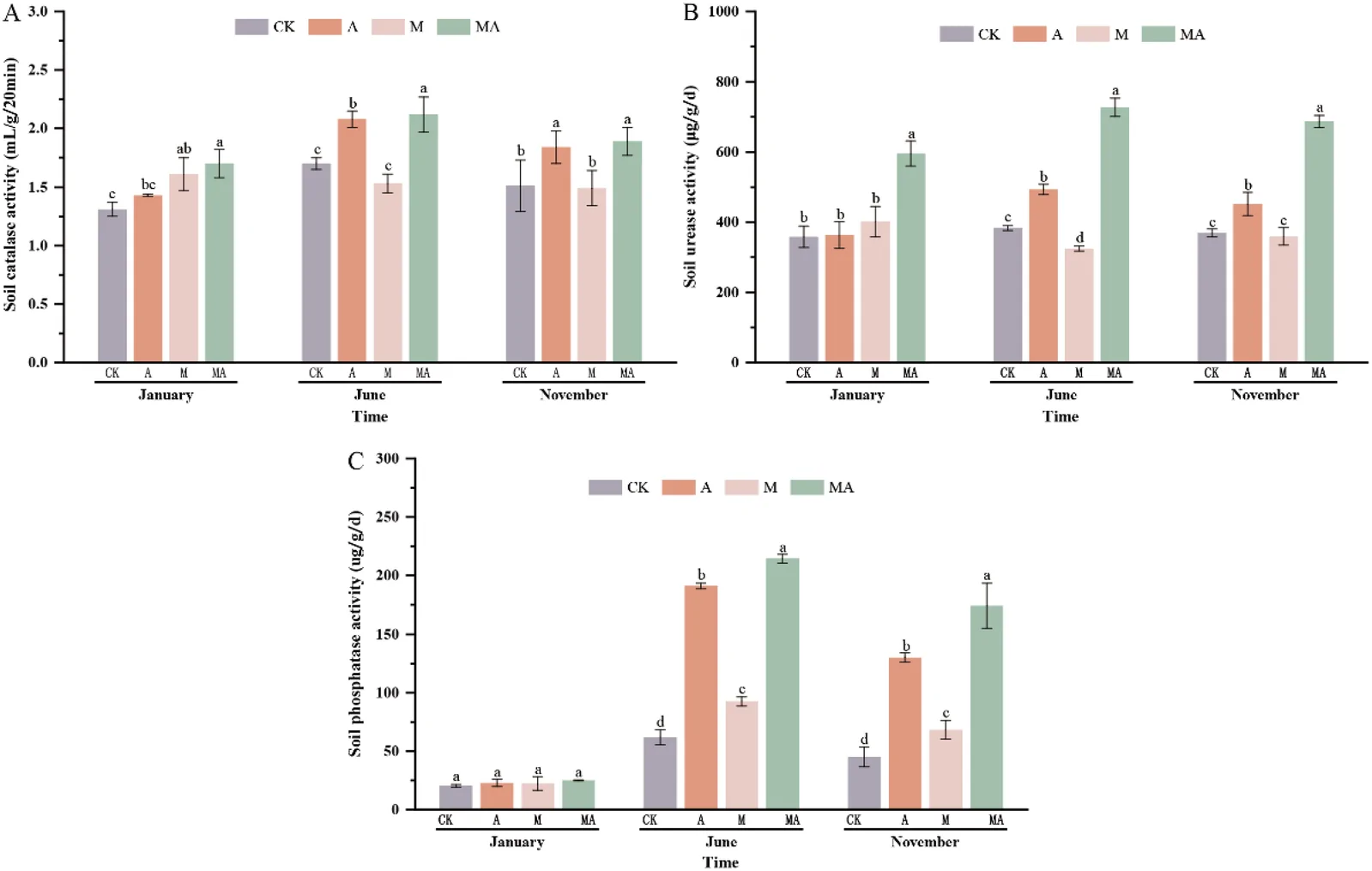
Effects of soil aeration on soil catalase **(A)**, urease **(B)**, and phosphatase **(C)** activity at different growth stages. The error bars represent the standard deviation (SD), n = 15. Different letters indicate significant differences between different treatments at the same sampling date (*p* < 0.05).

**Figure 4A** shows the catalase activity levels in the soil under different treatments during various growth periods. With the exception of the M treatment, catalase activity peaked during the rhizome development period in June. The lowest catalase activity for the CK and aerated treatments occurred in January, while for the M treatment, it was in November. Throughout the growth period, catalase activity in the aerated groups consistently exceeded that of the M and CK treatments, with MA being slightly higher than A (*p* > 0.05). In January, June, and November, catalase activity in the MA treatment was 5.59%, 38.56% (*p* < 0.05), and 26.85% (*p* < 0.05) higher than in the M treatment, respectively. Catalase activity in January, June, and November in the A treatment was 9.16%, 22.35% (*p* < 0.05), and 21.85% (*p* < 0.05) higher than in the CK treatment, respectively. Compared to January, catalase activity in the CK, A, and MA treatments increased in June by 29.77%, 45.45%, and 24.71%, respectively. In November, they increased by 15.27%, 28.67%, and 11.18%, respectively. In contrast, catalase activity in the M treatment decreased by 4.97% and 7.45% in June and November, respectively.

The urease activity in the soil during the different growth stages under various treatments is shown in **Figure 4B**. As the treatment period progressed, the aerated groups generally showed an increasing trend. Except for the M treatment, urease activity peaked during the rhizome development period in June. In January, June, and November, urease activity in the MA treatment was 48.18%, 124.08%, and 90.99% higher than in the M treatment, respectively. Urease activity in January, June, and November in the A treatment was 1.46%, 28.64%, and 22.11% higher than in the CK treatment, respectively. Compared to January, urease activity in the CK and A treatments in June increased by 6.98% and 3.12%, and in November by 35.64% and 24.10%, respectively, while urease activity in the M treatment decreased by 19.17% and 10.39%.

During the growth period, soil phosphatase activity in all treatments initially increased and then decreased (**Figure 4C**), peaking in June (rhizome tillering stage). In January, June, and November, phosphatase activity in the MA treatment was 11.55%, 131.71%, and 155.22% higher than in the M treatment, respectively. Phosphatase activity in January, June, and November in the A treatment was 11.99%, 208.80%, and 187.07% higher than in the CK treatment, respectively. Compared to January, phosphatase activity in the CK, A, M, and MA treatments increased by 203.08%, 121.44%, 735.71% and 467.61% in June, and by 314.6%, 205.69%, 761.18% and 599.36% in November, respectively.

### 3.3 Effects of aeration on physiological indices of Lei bamboo roots

**Figure 5:**
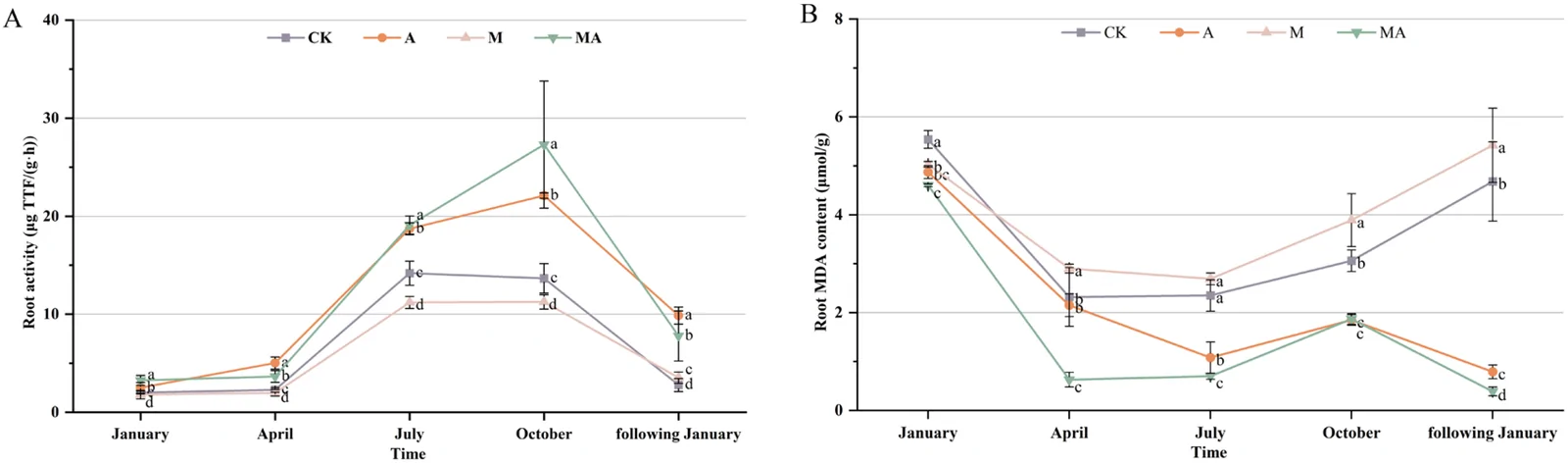
Effects of soil aeration on root activity [**(A)**, n = 45] and MDA content [**(B)**, n = 18] of Lei bamboo at different growth stages. The error bars represent the standard deviation (SD). Different letters indicate significant differences between different treatments at the same sampling date (*p* < 0.05).

During the experimental period, the root activity trends in the four treatments were consistent, all displaying a single-peak curve (**Figure 5A**). Differences between treatments began to increase in July, reaching a maximum in October (post-tillering root maturation stage). In October, root activity in the MA treatment was 142.67% higher than M, and activity in A was 61.90% higher than CK. The root activity in the aerated treatments peaked in October, while the non-aerated treatments peaked in July. Compared with January, the root activity of MA and A increased by 740.00% and 139.38% in October and by 784.00% and 294.40% the following January, respectively. For CK and M, the increases were 605.47% and 38.31% in July and 522.22% and 95.56% the following January, respectively.

The changes in the root MDA content of Lei bamboo during different growth stages are shown in **Figure 5B**. Over the course of a year, the trends in root MDA content varied among the four treatments. The non-aerated treatments (M and CK) followed a single-peak curve. The M treatment peaked in September, while all other treatments peaked in January. Compared with the initial values recorded in January, one year of aeration resulted in significant differences in root MDA content among the treatments. The CK, A, and MA treatments showed decreases of 15.52%, 83.78%, and 91.52%, respectively, while the M treatment exhibited an increase of 7.75%.

**Figure 6:**
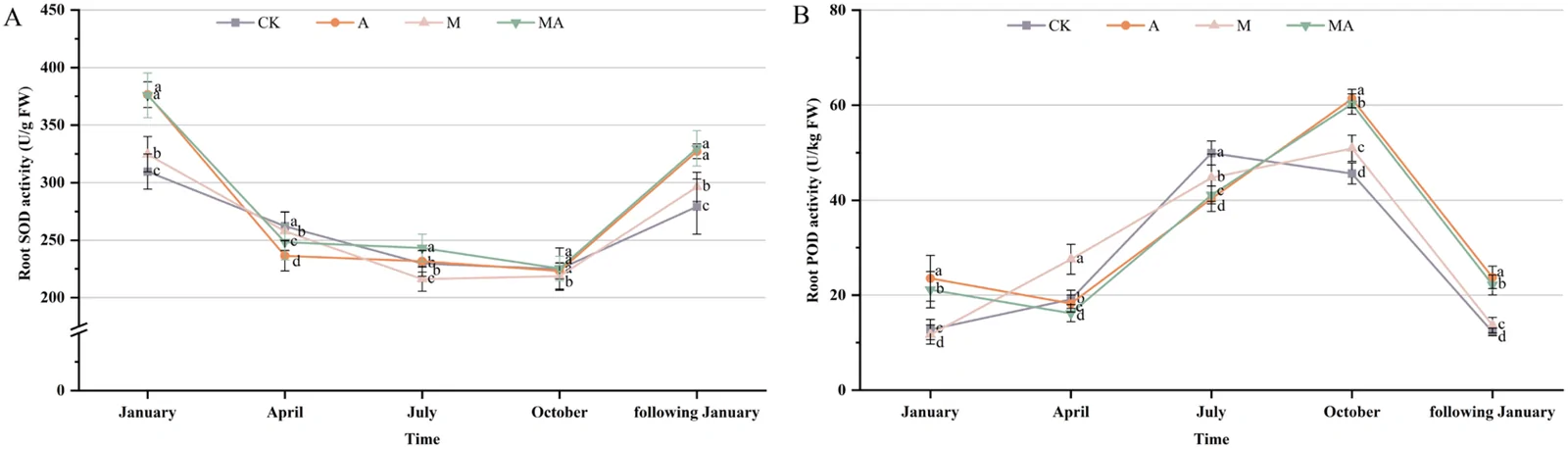
Effects of soil aeration on root SOD **(A)** and POD **(B)** activity of Lei bamboo at different growth stages. The error bars represent the standard deviation (SD), n = 18. Different letters indicate significant differences between different treatments at the same sampling date (*p* < 0.05).

During the experimental period, the root SOD activity in the four treatments exhibited a V-shaped curve (**Figure 6A**). The aerated treatments (MA and A) and the CK treatment reached their lowest values in October, at 225.26 U/kg FW, 223.14 U/kg FW, and 224.73 U/kg FW, respectively, while the SOD activity in the M treatment reached its lowest value in July (216.11 U/kg FW). After one year of aeration, the root SOD activity of the MA, A, CK, and M treatments decreased by 12.25%, 13.08%, 9.84%, and 8.72%, respectively.

With the extension of the treatment period, the changes in POD activity in the roots of the aerated group and the non-aerated group differed (**Figure 6B**). In the aerated group (MA and A), POD activity decreased initially, then increased, and finally decreased, while the non-aerated group (CK and M) exhibited a single-peak curve. The POD activity of the aerated group and the M treatment reached their highest values in October, whereas the CK treatment peaked in July at 49.94 U/kg FW. Significant differences in POD activity were observed among treatments in April and October. Compared to January, the POD activity of MA and A decreased by 23.38% and 22.52% in April, while that of CK and M increased by 49.88% and 135.38%, respectively. Compared to April, the POD activity of MA, A, CK, and M increased by 272.02%, 236.64%, 138.78%, and 84.89% in October, respectively. In April, MA was 41.21% lower than M, and A was 4.60% lower than CK. In October, MA was 18.28% higher than M, and A was 34.49% higher than CK.

### 3.4 Effects of aeration on the leaf photosynthetic rate

**Figure 7:**
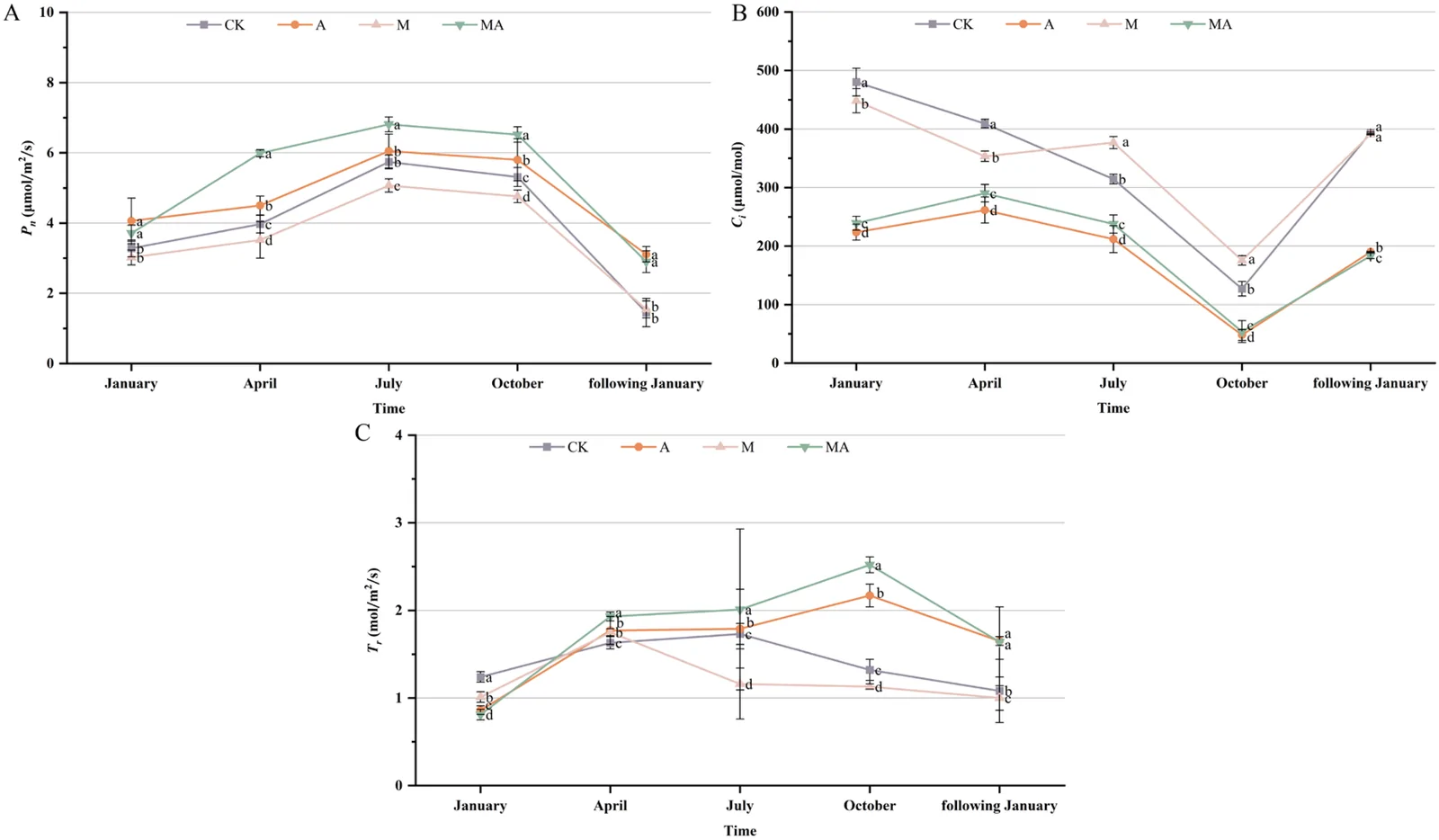
Effects of soil aeration on the photosynthetic rate (*Pn*) **(A)**, intercellular CO_2_ concentration (*Ci*) **(B)**, and transpiration rate (*Tr*) **(C)** of Lei bamboo leaves. The error bars represent the standard deviation (SD), n = 18. Different letters indicate significant differences between different treatments at the same sampling date (*p* < 0.05).

Over the course of the year, the leaf photosynthetic rate of MA, A, CK, and M treatments first increased and then decreased, with the aerated group consistently higher than the non-aerated group (**Figure 7A**). The leaf photosynthetic rate reached its peak in July (summer, maximum light/temperature) for all treatments. Compared to January, these represented increases of 83.56%, 49.01%, 75.00%, and 67.88%, respectively. In January, the photosynthetic rate in the MA treatment was 22.85% higher than M, and the rate in the A treatment was 23.78% higher than CK. After one year of aeration, the rate in the MA treatment was 88.31% higher than M, and A was 114.48% higher than CK.

The variation in intercellular CO_2_ concentration in the leaves under different treatments at various growth stages is shown in **Figure 7B**. In the aerated groups, the intercellular CO_2_ concentration first increased and then decreased, while the CK group showed a continuous decline, and the CO_2_ concentration in the M treatment first declined and then increased. The minimum values for the CO_2_ concentrations in the MA, A, CK, and M treatments were recorded in October, and these values represented a decrease of 77.43%, 78.51%, 73.54%, and 60.86% compared to January. In comparison to April, the intercellular CO_2_ concentrations in July had decreased by 18.14%, 19.01%, and 23.14% for the MA, A, and CK treatments, respectively, while the M treatment had increased by 6.61%.

The transpiration rates all exhibited an initial increase followed by a decline, though the rates of increase and the timing of peak values varied among treatments (**Figure 7C**). On a temporal scale, differences among treatments were relatively small in January, with the transpiration rate of the MA treatment being 19.80% lower than that of the M, and the A treatment being 30.65% lower than CK. By July, likely due to high temperatures, no significant differences were observed among treatments, and larger errors were noted. In contrast, the differences among treatments were most pronounced in October, with the transpiration rate of the MA being 123.01% higher than that of the M, and the A being 62.12% higher than CK. Compared to January 2023, the transpiration rates in October increased by 211.11%, 148.84%, 39.52%, and 73.27% for the MA, A, CK, and M treatments, respectively. After one year of aeration, the transpiration rates of the MA and A treatments increased by 102.50% and 91.86%, respectively, while the CK and M treatments decreased by 12.90% and 0.99%, respectively.

### 3.5 Effects of soil aeration on soluble protein and soluble sugar content of the leaves

**Figure 8:**
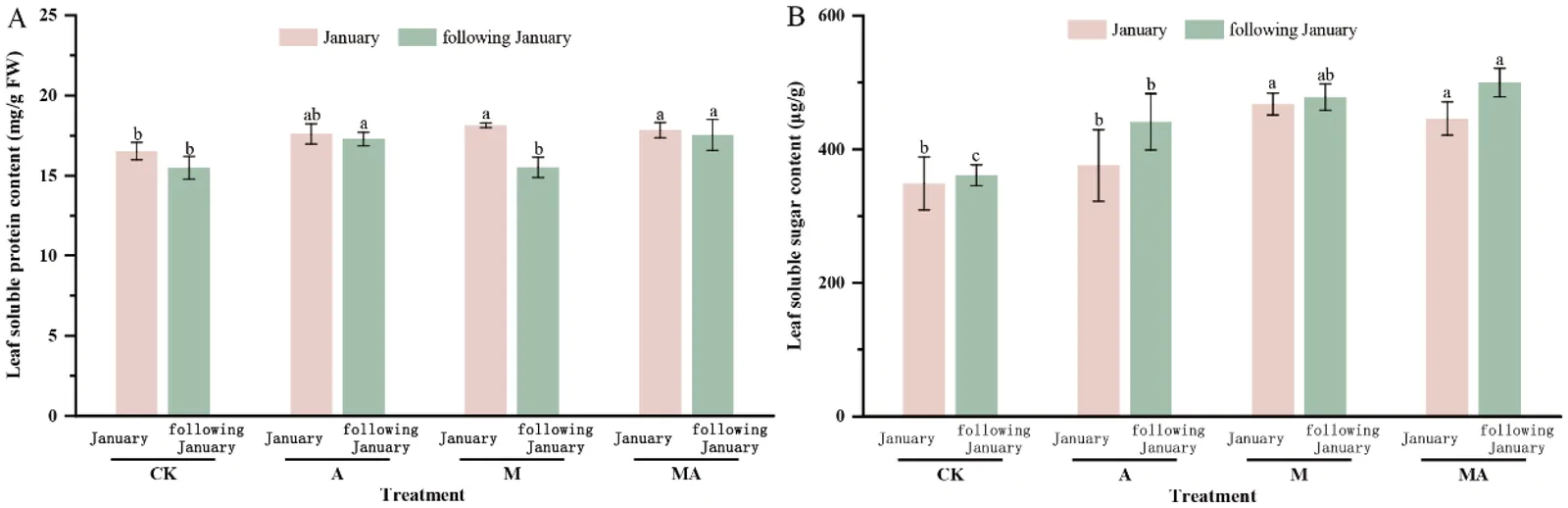
Effects of soil aeration on the soluble protein **(A)** and soluble sugar **(B)** content of Lei bamboo leaves. The error bars represent the standard deviation (SD), n = 18. Different letters indicate significant differences among treatments at the same sampling date (*p* < 0.05).

**Figure 8** illustrates the trends in leaf soluble protein and soluble sugar content across treatments before and after one year of aeration treatment. At the initial trial stage, both soluble protein and soluble sugar content were highest in the M treatment and lowest in the CK treatment, with no significant differences observed between CK and A, or between M and MA. After one year, at the end of the experiment, the soluble protein content in all treatments had decreased compared to the initial level (**Figure 8A**). The reductions were 1.79%, 1.71%, 6.29%, and 14.45% for the MA, A, CK, and M treatments, respectively. At this stage, the aeration treatments (MA and A) significantly higher soluble protein content than non-aerated treatments (CK and M). In contrast, the soluble sugar content in all treatments had increased (**Figure 8B**), with increases of 12.11%, 17.41%, 3.49%, and 2.25% for the MA, A, CK, and M treatments, respectively, and the aeration treatments (MA and A) showing markedly higher levels than the non-aeration treatments (CK and M).

## 4 Discussion

### 4.1 Effects of soil aeration on soil enzyme activity

Soil enzymes are important drivers of the transformation of carbon, nitrogen, phosphorus, sulfur, and other elements in the soil, and reflect the growth and development status of plants as well as their nutrient utilization capacity (Bandick and Dick, 1999). We found that soil enzyme activity in Lei bamboo varied significantly over time. The dynamics of the different enzyme activities during different growth periods were similar, except in the M treatment. Enzyme activity generally followed a trend of first increasing and then decreasing, peaking during the rhizome tillering stage in June (**Figure 4**). This pattern is consistent with studies of other crops, such as tomatoes (Niu et al., 2012b). This may be because soil enzymes are secreted by plant roots and rhizosphere microorganisms, and the abundance of rhizosphere microorganisms is influenced by root activity (Li et al., 2016). In the early growth season for Lei bamboo, energy and nutrients for microbial growth are abundant, oxygen is sufficient, and root metabolism is vigorous, leading to increased root exudates and a continuous rise in soil enzyme activity. In the later stages, as the roots deplete these resources, oxygen and soil nutrients decrease, reducing the substrate for soil enzyme activity and weakening enzyme function.

Soil enzyme activity is influenced by various factors such as the water, temperature, and air in the soil, and by soil texture. In this study, soil enzyme activity in the aerated treatments was consistently higher than in the non-aerated group (**Figure 4**). This suggests that aeration significantly enhanced the activity of soil catalysis, urease, and phosphatase, with the most significant difference observed during the tillering stage of the bamboo. This is likely due to the direct improvement of air conditions in the root zone soil by aeration, which increased the oxygen concentration in the soil and, during the summer, helped reduce soil temperature (Qian et al., 2022b). These factors directly influence the soil microbial community and soil fertility, enhancing microbial activity (Zhou Y et al., 2020), thereby increasing soil enzyme activity. Comparison of the soil enzyme activity in the MA and A treatments across different periods showed that the MA treatment exhibited significantly higher soil enzyme activity than the A treatment, although the difference was small. This suggests that under a mulch, aeration further enhances soil enzyme activity. This may be because the cover improves the physical and chemical properties of the soil, increases microbial population and activity, and thus enhances enzyme activity, which is consistent with previous studies (Qian et al., 2015).

### 4.2 Effects of soil aeration on the root system of Lei bamboo

The plant root system is important for supplying water and nutrients to the plant, and its growth and development directly affect the growth of the aerial parts. During winter, the root activity of Lei bamboo was relatively low under all treatments (**Figure 5A**). This is because plants are typically more sensitive to soil temperature than to air temperature. At the beginning of the experiment, root activity in the aerated treatments was slightly higher than in the non-aerated treatments. As spring arrived, root activity increased across all treatments, but no significant differences were observed between the aerated and non-aerated treatments, possibly because soil temperature was still the primary factor influencing root activity in early spring. From July to October, as temperatures warmed, the effect of aeration on root activity gradually increased. Activity in the aerated treatments continued to rise and reached their peak, while root activity in the non-aerated treatments declined. After one year of aeration, compared to January 2023, root activity in January 2024 was significantly higher, with activity in the MA treatment being slightly lower than in the A treatment. Throughout the entire growth period, significant differences in root activity were observed between the different treatments, with aerated treatments consistently showing higher root activity than non-aerated treatments. These results are consistent with the research conducted on tomato (Niu et al., 2012a; Wang et al., 2025), cotton (Qi et al., 2016) and peach trees (Sun et al., 2022).

MDA is an active aldehyde produced by lipid peroxidation under oxidative stress. High MDA levels are an indicator of increased membrane lipid peroxidation and oxidative stress (Masood et al., 2024), reflecting the extent of oxidative damage in plants. Studies have shown that compared to non-aerated conditions, MDA content in tomato plants is significantly reduced under aerated drip irrigation conditions (Zhu et al., 2022). We obtained a similar result, with the difference in MDA content between the aerated group and the non-aerated group increasing over time. In the early stages of the experiment (from January to April), the MDA content in roots in the aerated treatments was significantly reduced (**Figure 5B**). In April, as soil temperature rose and roots became active, the MDA content in the M treatment was significantly higher than in the other treatments, suggesting that the hypoxic conditions created by the mulching were beginning to affect the roots. By the time of tillering, as root growth increased and oxygen demand rose, aeration alleviated root hypoxic stress, effectively reducing MDA accumulation. Throughout the growing season, MDA levels were significantly higher in the CK treatment during the winter, likely because the roots faced more severe stress during this period. Low-temperature stress disrupts root ROS homeostasis, leading to increased MDA levels, a response that has previously reported from Chinese crab apple trees *(Malus baccata*) (Zhao et al., 2024) and bottle gourds (*Lageraria siceraria*) (Liu et al., 2024), amongst many others. Other studies have not found this seasonal variation: In *Ipomoea pes-caprae*, MDA levels showed no significant seasonal differences (Jin et al., 2022), which may be due to differences in stress resistance mechanisms between species or different plant tissues or to the temperatures involved. In high-temperature environments, plant cell membranes may be damaged, leading to an increase in MDA content. This could be the reason for the slight increase in MDA levels in the aerated groups during the summer high-temperature period (July to October), but it remained much lower than in the non-aerated groups. From October 2023 to January 2024, MDA content in the non-aerated groups increased, whereas in the aerated groups, MDA content decreased, further demonstrating that aeration significantly reduced the accumulation of MDA caused by low-temperature stress in winter.

Under normal growth conditions, the production of free radicals in plants can be balanced by the antioxidant defense system (Hasanuzzaman et al., 2020). However, under stress, the accumulation of ROS disrupts this balance, leading to oxidative stress. SOD and POD are the key antioxidant enzymes in the ROS scavenging system and play an important role in protecting plants from oxidative damage induced by stress (Yang et al., 2019). During the entire growing season, SOD levels were higher in winter (**Figure 6**), likely because low-temperature stress caused excessive accumulation of ROS in the roots, activating the antioxidant system to remove ROS. SOD catalyzes the dismutation of superoxide anions into oxygen and hydrogen peroxide, which are then converted by catalase into harmless products (H_2_O and O_2_) to mitigate stress (Zhou et al., 2022), which is consistent with the findings on naked oats (*Avena nuda* L.) SOD resistance to low-temperature stress (Liu et al., 2013). POD activity was higher in summer and lower in winter, which may be due to POD acting during resistance to high temperature stress (Qian et al., 2024). The SOD activity in aerated treatments was consistently higher than non-aerated treatments, except April. Additionally, the POD activity initially decreased, and during April and July, it was lower than in the non-aerated groups. Based on the changes in MDA content, this could be because after a period of aeration, the bamboo in the MA and A treatments experienced less stress, resulting in a weaker antioxidant response and lower SOD activity.

The impact of aeration on SOD activity was most evident in winter, with significant differences between the aerated groups and non-aerated groups, suggesting that aeration could enhance SOD activity and improve the plant’s ability to cope with regional stress environments during winter. From July to October, POD activity in the aerated groups increased significantly, while in the CK treatment, POD activity decreased, and in the M treatment, it increased slightly. When compared to the MDA content during this period, the increased lipid peroxidation in the root membranes suggests that aeration significantly enhanced POD activity under high temperatures. This is consistent with the findings of increased POD activity in papaya (*Carica papaya* L.) fruit under oxygenation treatment (Feng et al., 2022). The trends in SOD and POD activities in this study were not perfectly synchronized, indicating a reciprocal relationship between these two antioxidant enzymes, with overall antioxidant enzyme activity remaining stable. However, some studies have shown that in plants like *Buxus sinica*, SOD and POD levels maintain similar trends over time, likely due to differences in function and environmental sensitivity between plant tissues, such as temperature and moisture responses. For example, heat-tolerant tissues may show higher antioxidant activity compared to temperature-sensitive tissues (Qian et al., 2024), leading to differences in the coordination mechanisms of antioxidant enzymes.

### 4.3 Effects of soil aeration on the leaves of Lei bamboo

The photosynthetic capacity and efficiency of leaves are among the most critical factors affecting the total photosynthetic output of crops (Ouyang et al., 2021). In our study, the leaf photosynthetic rate of Lei bamboo under all treatments first increased and then decreased during the entire growth period, peaking in July (**Figure 7**). This can be attributed to low temperatures, weak light intensity, and short daylight hours in winter, which limit photosynthesis. In summer, as temperatures rise and light intensity strengthens, energy and material exchanges between leaves and the external environment accelerate (Xie et al., 2024; Jiang et al., 2024). This seasonal pattern is not universal across plant species: The net photosynthetic rate of Chinese fir (*Cunninghamia lanceolata* (Lamb.) Hook.) needles in summer has been documented as being significantly lower than in spring or autumn (Zhang Y et al., 2024), suggesting that different species respond differently to seasonal variations. As bamboos and Chinese fir are C3 plants, the results suggest that differences are not the result of the photosynthetic pathway. From January to April, the photosynthetic rate in the MA treatment increased significantly more than in other groups, potentially because the combination of aeration and mulching had an insulating effect, enhancing the photosynthetic capacity of Lei bamboo leaves. This is also consistent with an increase in photosynthesis with temperature in 11 temperate tree species (Reich et al., 2018). Throughout the growth period, the net photosynthetic rate of Lei bamboo leaves in the aerated treatments was significantly higher than in the non-aerated group. Even in winter, when photosynthesis weakened, the aerated treatments still exhibited higher photosynthetic rates, suggesting that aeration significantly improved leaf photosynthesis during the winter.

Carbon dioxide is a key substrate for photosynthesis, and lower intercellular CO_2_ concentrations indicate higher photosynthetic rates and stronger CO_2_ utilization. In our study, the transpiration rate and net photosynthetic rate of CK-treated leaves showed opposite trends, consistent with the photosynthetic characteristics reported for *Larix gmelinii* (Guan et al., 2024) and *Litchi chinensis* (Zhou R et al., 2020). Unlike CK, intercellular CO_2_ concentrations in aerated treatments increased from January to April, likely due to enhanced root respiration under higher oxygen, increasing rhizosphere CO_2_ diffusion to leaves.

Both transpiration and photosynthesis in leaves are influenced by stomatal conductance. During the growth period, leaf transpiration rates first increased and then decreased, consistent with trends observed in maize (Jiang et al., 2024) but opposite to those in *Cunninghamia lanceolata* (Zhang Y et al., 2024). This difference may be due to varying water requirements across species and growth stages, affecting stomatal conductance. The timing of peak transpiration rates varied among treatments: the aerated treatments peaked in October, CK in July, and M in April. Notably, peak transpiration rates in the aerated treatments were significantly higher than in the non-aerated group, indicating that aeration significantly enhanced leaf transpiration in Lei bamboo.

Lee et al. (2025) have suggested that net photosynthetic rates and transpiration rates of leaves are positively correlated. The trends in leaf transpiration rate and net photosynthetic rate in this study were almost identical, while they were inversely related to intercellular CO_2_ concentrations. This relationship is supported by research on maize (Jiang et al., 2024). Our results indicated the net photosynthetic rate was highest in July, whereas intercellular CO_2_ concentration and transpiration rates reached their lowest and highest values in October, respectively. The lack of synchronization between net photosynthesis, transpiration, and intercellular CO_2_ concentrations may arise because photosynthesis and transpiration, although regulated by guard cells, operate relatively independently.

Soluble proteins and soluble sugars in leaves are key indicators of photosynthetic capacity and material transport. Soluble proteins, which include enzymes involved in various metabolic processes such as Rubisco (accounting for 30–50% of soluble protein), are closely related to photosynthesis. Higher soluble protein contents indicate stronger photosynthetic capacity (Chen et al., 2007). Soluble sugars, photosynthetic products, also enhance plant stress tolerance (Zhou R et al., 2020). After one year of aeration, the decline in soluble protein content in the aerated treatments was significantly slower than in the non-aerated group. Additionally, soluble sugar content increased significantly more in the aerated treatments than in the non-aerated group (**Figure 8**). These results suggest that aeration delays leaf senescence, reduces the decline in soluble protein, and enhances the accumulation of soluble sugars. This finding is consistent with research showing that soluble sugar and protein content in pea (*Pisum sativum* L.) increased in aerated treatments compared to CK (Sayed and Gadallah, 2019). However, in January 2023, no significant differences were observed between M and the aerated treatments (**Figure 8**), possibly because mulching in the early stages of the experiment influenced soluble protein content by retaining soil moisture (Zhou and Shen, 2013), indirectly affecting plant growth. Among three winter wheat varieties grown in soils of different textures, soluble protein content in flag leaves during the grain-filling stage also varied. After one year of aeration, significant differences in soluble protein content were observed between the aerated treatments and the non-aerated group, demonstrating the significant impact of aeration on protein changes. In January 2023, the soluble sugar content in mulched group (M and MA treatment) was significantly higher than in non-mulching group (A and CK treatment). After one year, MA differed from M but not significantly, while MA differed significantly from A. Soluble sugar contents in MA, M, and A treatments were notably higher than in CK. This could be due to improved soil nutrient conditions under mulching, which promoted the accumulation of soluble sugars. This finding aligns with the results that plastic film mulching could maintain the normal level of soluble sugar in wheat under stress environment (Irshad et al., 2021).

## 5 Conclusions

Soil hypoxia induced by intensive mulching is the primary driver of Lei bamboo forest degradation and a serious threat to the sustainability of plantations. To address this issue, this study introduced buried soil pipes aeration technology into a plantation. The results demonstrate that this technique not only effectively elevates soil oxygen content without altering soil temperature, but also ensures normal plant growth and development by enhancing rhizosphere soil enzyme activity and root vitality, mitigating oxidative stress and improving photosynthetic efficiency in mulched Lei bamboo. This clean technology provides an innovative solution for combating degradation in mulched Lei bamboo forests, successfully achieving the dual objectives of off-season production and sustainable management. Additionally, interannual validation of the technique’s application efficacy further confirms its adaptability to varying climatic conditions. Collectively, the research delivers an evidence-based, practical and sustainable way to offset the adverse effects of current management practices on Lei bamboo forests. The technology has potential applications in the management of oxygen stress in other high-value perennial crops, resulting in cleaner, more sustainable, agricultural production.

## Data Availability

The original contributions presented in the study are included in the article/supplementary material. Further inquiries can be directed to the corresponding author.
